# Determinates of *Clostridioides difficile* infection (CDI) testing practices among inpatients with diarrhea at selected acute-care hospitals in Rochester, New York, and Atlanta, Georgia, 2020–2021

**DOI:** 10.1017/ice.2022.205

**Published:** 2023-07

**Authors:** Scott K. Fridkin, Udodirim N. Onwubiko, William Dube, Chad Robichaux, Jessica Traenkner, Dana Goodenough, Frederick J. Angulo, Joann M. Zamparo, Elisa Gonzalez, Sahil Khanna, Christopher Myers, Ghinwa Dumyati

**Affiliations:** 1 Division of Infectious Diseases, Department of Medicine, Emory University, Atlanta, Georgia; 2 Rollins School of Public Health, Emory University, Atlanta Georgia; 3 Division of Biomedical Informatics, Department of Medicine, Emory University, Atlanta, Georgia; 4 Foundation for Atlanta Veterans’ Education and Research, Decatur, Georgia; 5 Atlanta Veterans’ Affairs Medical Center, Decatur, Georgia; 6 Georgia Emerging Infections Program, Atlanta, Georgia; 7 Medical Development and Scientific/Clinical Affairs, Pfizer Vaccines, Collegeville, Pennsylvania; 8 Division of Gastroenterology and Hepatology, Mayo Clinic, Rochester, Minnesota; 9 Center for Community Health and Prevention, University of Rochester Medical Center, Rochester, New York; 10 Infectious Diseases Division, University of Rochester Medical Center, Rochester, New York

## Abstract

**Objective:**

We evaluated the impact of test-order frequency per diarrheal episodes on *Clostridioides* difficile infection (CDI) incidence estimates in a sample of hospitals at 2 CDC Emerging Infections Program (EIP) sites.

**Design:**

Observational survey.

**Setting:**

Inpatients at 5 acute-care hospitals in Rochester, New York, and Atlanta, Georgia, during two 10-workday periods in 2020 and 2021.

**Outcomes:**

We calculated diarrhea incidence, testing frequency, and CDI positivity (defined as any positive NAAT test) across strata. Predictors of CDI testing and positivity were assessed using modified Poisson regression. Population estimates of incidence using modified Emerging Infections Program methodology were compared between sites using the Mantel-Hanzel summary rate ratio.

**Results:**

Surveillance of 38,365 patient days identified 860 diarrhea cases from 107 patient-care units mapped to 26 unique NHSN defined location types. Incidence of diarrhea was 22.4 of 1,000 patient days (medians, 25.8 for Rochester and 16.2 for Atlanta; *P* < .01). Similar proportions of diarrhea cases were hospital onset (66%) at both sites. Overall, 35% of patients with diarrhea were tested for CDI, but this differed by site: 21% in Rochester and 49% in Atlanta (*P* < .01). Regression models identified location type (ie, oncology or critical care) and laxative use predictive of CDI test ordering. Adjusting for these factors, CDI testing was 49% less likely in Rochester than Atlanta (adjusted rate ratio, 0.51; 95% confidence interval [CI], 0.40–0.63). Population estimates in Rochester had a 38% lower incidence of CDI than Atlanta (summary rate ratio, 0.62; 95% CI, 0.54–0.71).

**Conclusion:**

Accounting for patient-specific factors that influence CDI test ordering, differences in testing practices between sites remain and likely contribute to regional differences in surveillance estimates.

The Centers for Disease Control and Prevention (CDC) estimates that each year there are ∼462,000 episodes of *Clostridioides difficile* infection (CDI) occur in the United States each year, about half of which occur in patients presenting to or being cared for in hospitals.^1^ Estimates of the hospital-based CDI disease burden are commonly based on claims data or public health surveillance of laboratory-confirmed infections. Regardless of the methodology used, the estimation depends on several steps in the CDI laboratory testing pathway including preanalytic steps and analytic steps (eg, sensitivity of the test).^2^ Globally these estimates vary greatly; in general, higher incidences are observed in US-based studies, likely due to relatively higher use of more sensitive tests.^3^ Previous studies have focused on variations in sensitivity of specific diagnostic tests to explain differences in some CDI burden estimates,^4–7^ but few studies have evaluated the impact of test order frequency on differences in estimates of CDI incidence.^8,9^

The CDC Emerging Infections Program (EIP) conducts active population- and laboratory-based surveillance of all incident CDI in 10 geographically diverse parts the United States.^1^ The EIP evaluates all positive tests for *C. difficile* to identify CDI cases. Events leading to ordering each test (eg, decision to test, testing methods) depend on the individual standard-of-care practice in place at each clinic, hospital, or referral laboratory. Variations in such practices may influence estimated CDI incidences between surveillance sites.^10^ Some differences in the population-based CDI incidences between sites were noted; however, reasons for these differences have not been fully explored.^11^ We sought to determine whether differences in testing practice depend on patient characteristics and whether any differences in observed incidence of CDI through established surveillance methods reflect differences observed in adjusted testing rates.

## Methods

### Hospital selection

This observational cross-sectional study was conducted at 5 acute-care hospitals located in 2 EIP surveillance sites. Participants included 3 hospitals in Atlanta, Georgia, from a single healthcare system (hospitals 1–3 at site A), and 2 hospitals in Rochester, New York (hospitals 1 and 2 at site B). Nursing staff at each of the hospitals were oriented to the period-prevalence assessment process by study staff, and they identified efficient methods for reporting all diarrheal episodes (≥3 stools in 24 hours) during the study period. The study protocol was approved by the appropriate institutional review board at each study site. During the study period, each of the participating hospitals utilized NAAT either as a primary *C. difficile* test or as a secondary reflex test for a negative toxin enzyme immunoassay (EIA) with a positive glutamate dehydrogenase test. CDI testing practices were performed according to local protocols and practice, and each participating hospital had active diagnostic stewardship efforts in place to minimize unnecessary testing of stool specimens for *C. difficile*. The programs were similar, with an exception: 3 hospitals at site A included an educational effort to nursing staff to identify and suggest CDI testing to providers as an effort to test patients experiencing diarrhea upon of admission (Supplementary Table S1 online).

### Period-prevalence assessment

All inpatient adult patient-care units were eligible for participation and were approached for recruitment into the assessment, with an opt-out option if nursing staff shortages interfered with completing the activities. Patient-care units were defined by pre-existing mapping conventions for reporting to the CDC National Healthcare Safety Network (NHSN) to “location types.” We identified 2 distinct 10-day periods in the fall of 2020 and spring of 2021 (ie, 10 consecutive weekdays over a 14-day period) separated by at least 5 months at each facility to perform the prevalence assessment (Supplementary Table S1 online).

Study staff reviewed study processes with nursing staff prior to each assessment. On each assessment day, nursing staff recorded the bed number for each patient observed with diarrhea and stool sample order or collection status. Diarrhea was defined as having at least 3 unformed stools in the prior 24 hours, although no validation or confirmation of frequency and consistency was performed via chart review. Weekend diarrheal episodes were reported to Monday nursing staff to determine whether diarrhea frequency over the previous 24 hours was sufficient for reporting. For each diarrheal episode, limited characteristics of the patient and *C. difficile* testing results were captured from the electronic medical record. Characteristics included exposures potentially causing diarrhea such as laxatives, chemotherapy, and tube feedings through a nasogastric or percutaneous tube in the 48 hours before first day of diarrhea.^12–14^ Previous *C. difficile* testing history and positive severe acute respiratory coronavirus virus 2 (SARS-CoV-2) tests were limited to 14 days before the first day of diarrhea. For this analysis, we considered any positive test for *C. difficile* as a CDI-positive stool, which reflects the established surveillance methods of the EIP population-based estimates.

### Definitions and analytic approach

Diarrheal episodes were defined as the cumulative days a patient had diarrhea reported by nursing staff. Diarrheal episodes were classified as “pre-existing” if present prior to the first day of the period assessment or “new” if not present prior to the first day of the assessment. Episodes in patients with any CDI test ordered (regardless of result) in the previous 14 days were also considered pre-existing. New diarrheal episodes were classified as hospital onset if the first day of diarrhea occurred on or after the third day of hospitalization. To evaluate the influence of patient mix on testing practices, we aggregated data by NHSN patient-care type in each hospital. Hospital-based rates (per 1,000 patient days), stratified by site and patient-care location type (wards, oncology, and critical care) were calculated for new diarrheal episodes, hospital-onset episodes, and CDI positivity. Denominators were derived from the number of occupied beds per location per day of the observed study, without excluding any patient days among already CDI-positive patients. The percentage of episodes with CDI test ordered and the percentage of CDI tests positive for CDI, stratified by site and patient care location, were also calculated.

### Statistical analysis

Demographic and clinical characteristics of patients with new diarrheal episodes were characterized by study site, and differences in distribution were examined using the χ^2^ for categorical variables and the Kruskal-Wallis rank-sum test for continuous variables. Median NHSN unit-specific incidence across study sites, patient-care location types, and participating hospitals were calculated and compared using Kruskal-Wallis tests. Bivariate Poisson regression models with robust error variances were used to examine the unadjusted associations between the clinical and demographic characteristics. Forward selection was used to identify significant correlates of CDI ordering and positivity from among the patient demographic and clinical characteristics.

Among the 860 participants included in the analytic sample, 8.4% were missing race, 2.9% were missing age, and 1.7% were missing jurisdiction of residence. Missing data are a potential source of bias.^15^ Therefore, multiple imputations using chained equations were used to create 30 sets of the data set with imputed values for the missing data and to estimate pooled risk ratios.^16^

### Simulation of population-based estimates

We hypothesized that the magnitude of the difference in testing frequency between the study sites after adjusting for factors influencing likelihood to test (eg, diagnostic stewardship) would be reflected in any observed differences in EIP-derived estimates of CDI incidence. Because the participating hospitals represented only 28% of general acute-care hospitals in the relevant counties (22% of Dekalb–Futon and 50% of Monroe), we did not extrapolate testing practices countywide to avoid biased results. Therefore, we tested the hypothesis simulating EIP case finding using data from the 5 hospitals. We extracted positive *C. difficile* tests and admission data for a 12-month period that included both of our study periods. The data were limited to only patients residing in the surveillance catchment area of the respective EIP site and were stratified by age, race, and sex, similar to EIP.^1^ We further limited cases to samples collected during the hospital admission. The differences in age, race, and sex-specific CDI incidence were calculated between strata and were summarized using the Cochran–Mantel–Haenszel test.^16^

## Results

Among the 5 participating hospitals, 107 (96%) of the 112 patient-care units participated in at least one 10-day observation period: 71 (93.4%) of 76 at site A and 36 (94.7%) of 38 at site B. These 107 patient-care units accounted for 2,397 beds and 38,365 patient days and mapped to 26 unique NHSN-defined location types. Patient-care units were most commonly mapped to non–critical-care units: medical (n = 19), surgical (n = 12), oncology specialty (n = 12), medical-surgical combined (n = 11). Among critical care units, cardiothoracic (n = 7), medical (n = 6), coronary (n = 5), medical-surgical (n = 4), and neurosurgical (n = 4) were most common.

In total, 1,588 diarrheal episodes were identified, of which 860 (435 at site A and 425 at site B) were new diarrheal episodes (Supplementary Table S1 online). When comparing patient characteristics, patients in site A, compared to site B, were more likely to be Black (56.3% vs 20.2%), more likely to be aged <50 years (24.6% vs 17.6%), less likely to have received laxatives (33.3% vs 64.5%), more likely to have received chemotherapy (7.4% vs 1.9%), and more likely to have been discharged home (64.5% vs 31.0%). Of the patients with new diarrheal episodes, 62.4% were residents of the EIP surveillance areas (65.0% in site A and 59.8% in site B) (Table 1).

**Table 1.** Characteristics of Study Participants Patients With New Diarrheal Episodes: Period-Prevalence Survey

Characteristics	Overall (N = 860), No. (%)	Site A (N = 435), No. (%)	Site B (N = 425), No. (%)	*P* Value
**Race**				<.001^a^
Missing	72	55	17	
White	462 (58.6)	159 (41.8)	303 (74.3)	
Black	296 (37.6)	214 (56.3)	82 (20.1)	
Other	30 (3.8)	7 (1.8)	23 (5.6)	
**Age**				<.001^a^
Missing	25	25	0	
<50 y	176 (21.1)	101 (24.6)	75 (17.6)	
50–64 y	260 (31.1)	145 (35.4)	115 (27.1)	
65–74 y	205 (24.6)	89 (21.7)	116 (27.3)	
≥75 y	194 (23.2)	75 (18.3)	119 (28.0)	
**Had an ostomy**				.008^a^
No	830 (96.5)	427 (98.2)	403 (94.8)	
Yes	30 (3.5)	8 (1.8)	22 (5.2)	
**Tube feeding**				.007^a^
No	561 (65.2)	265 (60.9)	296 (69.6)	
Yes	299 (34.8)	170 (39.1)	129 (30.4)	
**Received laxatives^c^**				<.001^a^
No	441 (51.3)	290 (66.7)	151 (35.5)	
Yes	419 (48.7)	145 (33.3)	274 (64.5)	
**Received chemotherapy**				<.001^a^
No	820 (95.3)	403 (92.6)	417 (98.1)	
Yes	40 (4.7)	32 (7.4)	8 (1.9)	
**Diagnosed with COVID-19**				.021^a^
No	824 (95.8)	410 (94.3)	414 (97.4)	
Yes	36 (4.2)	25 (5.7)	11 (2.6)	
**Length of stay, d**				.990^b^
Missing	52	50	2	
Median (IQR)	12 (6–23)	12 (6–23)	12 (7–22)	
**Admission location**				.024^a^
Wards	501 (58.3)	239 (54.9)	262 (61.6)	
Critical care	245 (28.5)	142 (32.6)	103 (24.2)	
Oncology	114 (13.3)	54 (12.4)	60 (14.1)	
**Discharge disposition**				<.001^a^
Missing	81	72	9	
Home	363 (46.6)	234 (64.5)	129 (31.0)	
Hospital	89 (11.4)	29 (8.0)	60 (14.4)	
Long-term care^d^	221 (28.4)	36 (9.9)	185 (44.5)	
Died/Hospice	106 (13.6)	64 (17.6)	42 (10.1)	
**Resident of EIP catchment**				.116^a^
Missing	15	15	0	
Yes	527 (62.4)	273 (65.0)	254 (59.8)	
No	318 (37.6)	147 (35.0)	171 (40.2)	

Note. IQR, interquartile range; EIP, Emerging Infections Program.

a Pearson χ^2^ test.

b Kruskal-Wallis rank-sum test.

c Laxatives included docusate sodium, sennosides, polyethylene glycol 3350, biscodyl, fiber, lactulose, and/or psyllium.

d Long-term care included discharge to long-term care facilities, inpatient rehabilitation facility, or skilled nursing facility.

The hospital-based incidence of new diarrheal episodes was 22.4 per 1,000 patient days (28.9 at site A vs 18.4 at site B); 61.5% of episodes (57.9% in site A and 65.2% in site B) were classified as hospital onset (Table 2A). The proportion of new diarrhea episodes tested for CDI was 35.1%, and we detected a large difference in the proportion tested for CDI between the sites: 20.9% at site A versus 49.0% at site B. However, within each site, the proportions of episodes tested were similar between hospitals. The proportions of CDI tests that were positive (16.6%) for CDI were similar at both sites: 17.4% at site A and 14.6% at site B.

**Table 2a.** Overall Average Rates of Diarrhea Episodes (DE), *Clostridiodes difficile* Infection (CDI) Test Ordering and Positivity Per 1,000 Patients Days: Fall 2020 and Spring 2021 Period-Prevalence Survey

Location	Total PD	New DE		HO DE		CDI Test Ordering		CDI Positivity	
		No.	Rate^a^	No. (%)^b^	Rate^a^	No. (%)^b^	Rate^a^	No. (%)^c^	Rate^a^
Overall	38,365	860	22.4	529 (61.5)	13.8	302 (35.1)	7.9	50 (16.6)	1.30
Site A	23,643	435	18.4	252 (57.9)	10.7	213 (49.0)	9.0	37 (17.4)	1.56
Site B	14,722	425	28.9	277 (65.2)	18.8	89 (20.9)	6.0	13 (14.6)	0.88
Ward	26,900	501	18.6	303 (60.5)	11.3	162 (32.3)	6.0	29 (17.9)	1.08
Critical care	6,406	245	38.2	160 (65.3)	25.0	78 (31.8)	12.2	9 (11.5)	1.40
Oncology	5,059	114	22.5	66 (57.9)	13.0	62 (54.4)	12.3	12 (19.4)	2.37
Site A, hosp. 1	10,610	220	20.7	132 (60.0)	12.4	105 (47.7)	9.9	20 (19.0)	1.89
Site A, hosp. 2	8,171	117	14.3	69 (59.0)	8.4	57 (48.7)	7.0	7 (12.3)	0.86
Site A, hosp. 3	4,862	98	20.2	51 (52.0)	10.5	51 (52.0)	10.5	10 (19.6)	2.06
Site B, hosp. 1	10,290	318	30.9	212 (66.7)	20.6	64 (20.1)	6.2	9 (14.1)	0.87
Site B, hosp. 2	4,432	107	24.1	65 (60.7)	14.7	25 (23.4)	5.6	4 (16.0)	0.90

Note. PD, patient days; DE, diarrheal episodes; HO, hospital-onset; CDI, *Clostridiodes difficile* infection.

a All rates are per 1,000 patient days.

b Percentage of new DE cases.

c Percentage of CDI tests ordered.

**Table 2b.** Patient Care Locations and Rates (per 1,000 PD) of New Diarrheal Episodes (DE) and *Clostridiodes difficile* Infection (CDI) Test Ordering Among Hospitalized Patients, By Site, Patient Care Location, and Hospital, Fall 2020 and Spring 2021 Period Prevalence Survey

Location^a^	Total PD	No. NHSN Location Types	No. Units	New DE Cases				CDI Test Ordering		
				No.	Median Rate (IQR)	*P* Value^b^	No. (%)^c^	*P* Value^d^	Median Rate (IQR)^a^	*P* Value^b^
Overall	38,365	26	107	860	20.6 (12.9–33.1)		302 (35.1)		6.4 (2.5–12.3)	
Site A	23,643	20	71	435	16.2 (11.3–30.8)	.001	213 (49.0)	<.001	6.5 (2.3–16.3)	.216
Site B	14,722	19	36	425	25.8 (20.2–44.7)		89 (20.9)		5.0 (2.6–8.3)	
Wards	26,900	14	63	501	16.0 (10.5–23.8)	.000	162 (32.3)	<.001	4.4 (2.3–8.3)	.012
Critical care	6,406	8	32	245	33.1 (22.0–44.9)		78 (31.8)		8.2 (2.4–17.5)	
Oncology	5,059	4	12	114	20.1 (17.0–25.7)		62 (54.4)		11.6 (8.0–14.1)	
Site A, hosp. 1	10,610	14	36	220	20.0 (12.6–32.7)	.003	105 (47.7)	<.001	7.4 (2.3–17.3)	.625
Site A, hosp. 2	8,171	11	21	117	13.5 (9.3–17.1)		57 (48.7)		5.2 (1.7–9.3)	
Site A, hosp. 3	4,862	11	14	98	16.8 (11.4–27.9)		51 (52.0)		9.0 (3.1–16.6)	
Site B, hosp. 1	10,290	16	28	318	29.6 (21.6–45.9)		64 (20.1)		4.7 (3.0–8.9)	
Site B, hosp. 2	4,432	6	8	107	21.8 (17.7–24.9)		25 (23.4)		5.7 (2.6–7.2)	

Note. PD, patient days; NHSN, National Healthcare Safety Network; IQR, interquartile range.

a All rates are per 1,000 patient days.

b Kruskal-Wallis test;^2^

c Percentage of new DE cases;^1^

d Fisher exact test.

**Fig. 1.**
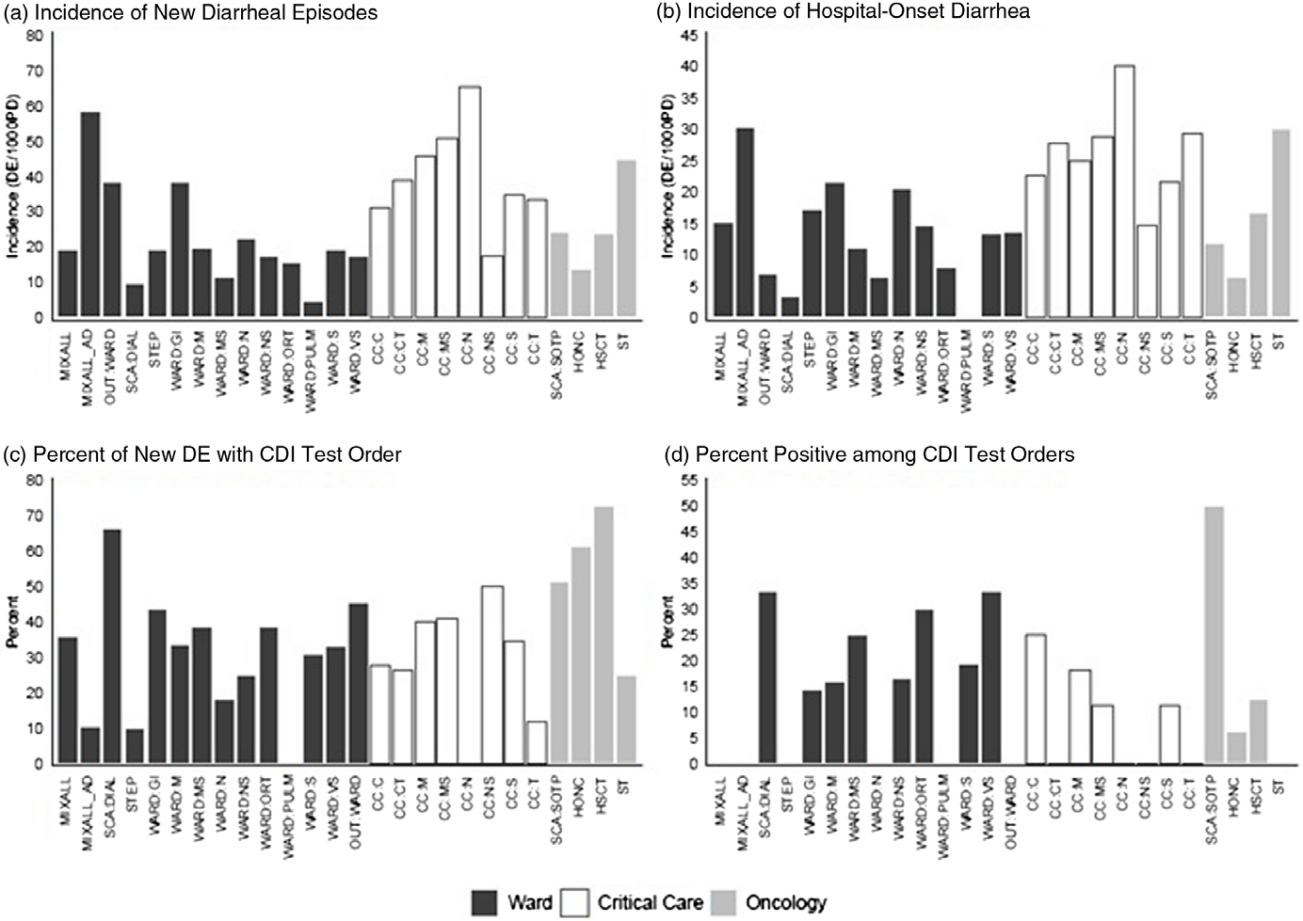
Incidence (per 1,000 patient days) of new diarrheal episodes (N = 860) and hospital-onset diarrhea, (N = 529), percentage of new diarrheal episodes with CDI test order (N = 302), and percentage of CDI test orders that tested positive (N = 50), among hospitalized patients, Fall 2020 and Spring 2021 period-prevalence survey. Note. CDI, *Clostridioides difficile* infection.

Hospital-based incidence (per 1,000 patient days) of new diarrheal episodes differed most dramatically between patient location types. It was highest among critical-care locations (33.1 per 1,000 patient days), then oncology (20.1 per 1,000 patient days), followed by other locations (16.0 per 1,000 patient days) (Table 2B and Fig. 1). The proportions of new diarrhea episodes tested for CDI also differed by patient care location, with highest rates among oncology locations (54.4%).

In univariate analysis, several factors were predictive of testing and were considered in the multivariable analysis (Table 3). In multivariable analysis, independent predictors of testing included care in an oncology location (adjusted RR, 1.57; 95% CI, 1.25–1.98), receiving laxatives (adjusted RR, 0.59; 95% CI, 0.48–0.74), and residing outside the surveillance catchment area (adjusted RR, 0.77; 95% CI, 0.62–0.94). Adjusting for these factors, patients with a diarrhea episode in site B were 49% less likely to receive a test than patients with diarrhea in site A (adjusted RR, 0.51; 95% CI, 0.40–0.63).

**Table 3.** Predictors of *Clostridiodes difficile* (CDI) Test Ordering: Fall 2020 and Spring 2021 Period-Prevalence Survey

Variable	No.	CDI Tests Ordered No. (%)	Unadjusted Regression Model		Adjusted Regression Model	
			RR (95% CI)	*P* Value	RR (95% CI)	*P* Value
**Race**						
White	462	144 (31.2)	Reference			
Black	296	120 (40.5)	1.30 (1.07–1.58)	0.008		
Other	30	7 (23.3)	0.75 (0.39–1.45)	.392		
**Age group**						
<50 y	176	67 (38.1)	Reference			
50–64 y	260	99 (38.1)	1.00 (0.78–1.28)	.999		
65–74 y	205	67 (32.7)	0.86 (0.65–1.13)	.272		
≥75 y	194	55 (28.4)	0.74 (0.56–1.00)	.048		
**Had an ostomy**						
No	830	295 (35.5)	Reference			
Yes	30	7 (23.3)	0.66 (0.34–1.26)	.208		
**Tube feeding**						
No	561	207 (36.9)	Reference			
Yes	299	95 (31.8)	0.86 (0.71–1.05)	.139		
**Received laxatives**						
No	441	211 (47.8)	Reference		Reference	
Yes	419	91 (21.7)	0.45 (0.37– 0.56)	<.001	0.59 (0.48–0.74)	<.001
**Received chemotherapy**						
No	820	278 (33.9)	Reference		Reference	
Yes	40	24 (60.0)	1.77 (1.35–2.32)	<.001	1.17 (0.89–1.55)	.262
**Diagnosed with COVID-19**						
No	824	287 (34.8)	Reference			
Yes	36	15 (41.7)	1.20 (0.80–1.78)	.377		
**Hospital-onset DE**						
No	308	125 (40.6)	Reference			
Yes	529	165 (31.2)	0.77 (0.64–0.92)	.005		
**Resident of catchment area**						
Yes	527	201 (38.1)	Reference		Reference	
No	318	94 (29.6)	0.78 (0.63–0.95)	.013	0.77 (0.62–0.94)	.011
**Admission location**						
Other (non–critical care)	501	162 (32.3)	Reference		Reference	
Critical care	245	78 (31.8)	0.98 (0.79–1.23)	.891	0.92 (0.73–1.15)	.452
Oncology	114	62 (54.4)	1.68 (1.36–2.08)	<.001	1.57 (1.25–1.98)	<.001
**Study site**						
Site A	435	213 (49.0)	Reference		Reference	
Site B	425	89 (20.9)	0.43 (0.35–0.53)	<.001	0.51 (0.40–0.63)	<.001

Note. RR, relative risk; CI, confidence interval.

Using testing results from these 5 hospitals among hospitalized residents of the catchment area to calculate age, race, and sex-specific incidence, both per 1,000 patient days and per 100 admissions, we detected lower hospitalized CDI rates in site B than site A for most strata (Fig. 3). Adjusting for age, race, and sex, the summary rate ratio of the hospitalized CDI rates at site B compared to site A was 0.62 (95% CI, 0.54–0.71), translating to ∼38% lower hospitalized CDI rate among residents of the EIP surveillance area identified from site B compared to site A.

**Fig. 2.**
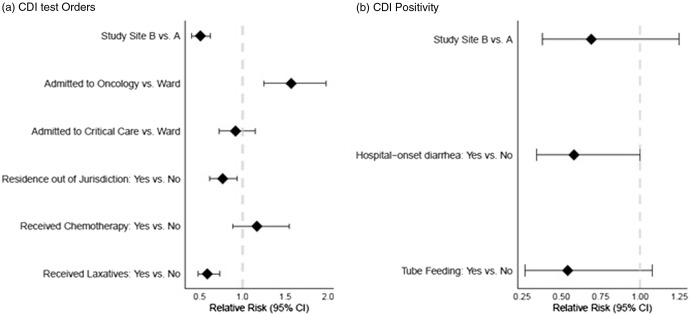
Period-prevalence survey (2020–2021). (A) Relative rate (and 95% confidence intervals) of selected characteristics for case with new diarrheal episode (N = 860) being CDI tested (N = 302). (B) For CDI test being positive (N = 50) among study participants. Note. CDI, *Clostridioides difficile* infection.

**Fig. 3.**
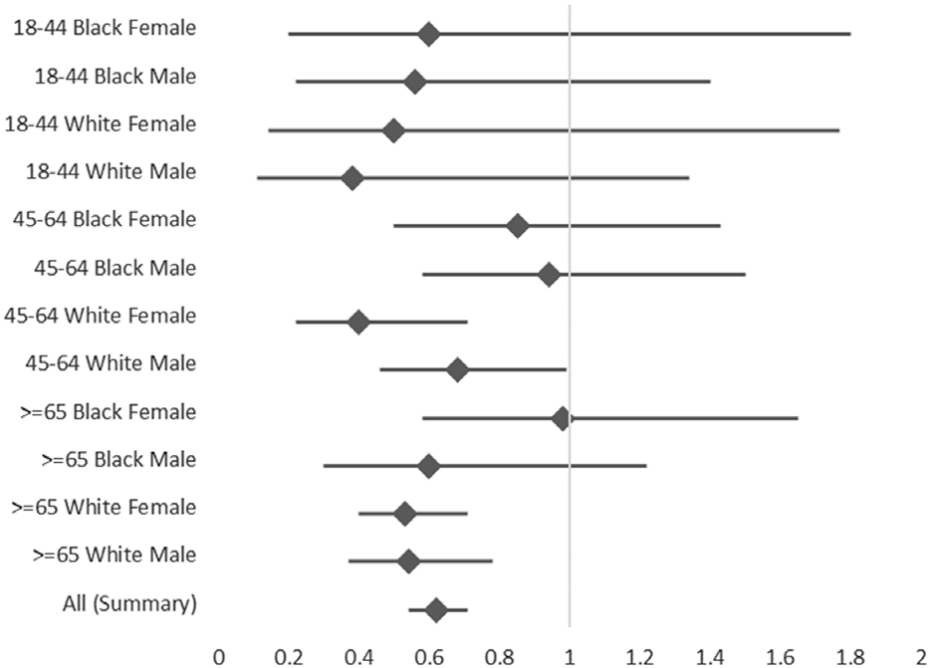
Crude and adjusted rate ratios of Monroe County residents compared to Atlanta area residents’ *C. difficile* incidence estimates calculated using retrospective simulation of Emerging Infection Program methods applied to inpatients at 5 hospitals, October 2020–September 2021. *Race data categorized as other race are not displayed but are included in summary rate ratio. Rate ratios compare rate (CDI positive diarrheal episodes among hospitalized residents per 100 admissions) in each age, race, and sex-specific stratum are crude calculations based on Taylor series. The “All” summary is a Mantel-Hanzel summary rate ratio adjusting for each stratum using OpenEpi version 3 software. Note. CDI, *Clostridioides difficile* infection.

## Discussion

Differences in testing frequency of diarrhea episodes for *C. difficile* among inpatients at acute-care facilities between 2 geographically distant locations persisted despite adjusting for patient factors that often influence decisions to test. The frequencies of testing inpatients with diarrhea were similar at the hospitals in each site, but they were an estimated 49% less frequent at the Rochester site compared to the Atlanta site, regardless of specific hospital. This observed difference was similar to the 38% lower CDI incidence estimated at the Rochester site compared to the Atlanta site using adjustments as done in the CDC EIP surveillance programs. These differences were essentially identical (within the 95% confidence intervals of each other), suggesting that testing practice may explain the difference in measured incidence observed through population-based surveillance for CDI.

Relying on *C. difficile* tests with high sensitivity, such as NAAT, might affect population rates CDI rates estimates,^8,9,17,18^ often leading to higher than true infection incidence because NAAT has lower specificity.^5,6,18,19^ However, our data suggest that differences in the intensity of testing patients, potentially driven by diagnostic stewardship, but definitely driven by differences in patient characteristics (eg, laxative use, oncology status), may also need to be considered when comparing incidence between regions. First, differences in *C. difficile* testing rates were profound even between the 2 sites, and they likely vary tremendously across diverse geographic areas in the United States and the world. Second, these differences in testing persisted after adjustment for differences in patient mix and potential alternative etiologies for diarrhea among inpatients. These differences may reflect penetration of messaging or programs to promote diagnostic stewardship within a healthcare system or region, or a more general culture of practice within a region. The reason for the differences is purely speculative; we did not systematically study them here.

Our study had several limitations. First, the testing practices between hospitals within each site may only vary minimally because of shared policies between facilities within each site. Also, the number of facilities participating in the study per site was small, reflecting a small proportion of all acute-care hospitals within the counties participating in population-based surveillance efforts for the EIP. This small sample size forced us to use an estimation method of CDI incidence limited to the 5 study hospitals. This study was conducted during the coronavirus disease 2019 (COVID-19) pandemic, which may have influenced testing practices in poorly understood ways. However, periods of study occurred during moderate nadirs in the COVID-19 pandemic, and we considered COVID-19 in the predictive model. Finally, excluding weekends likely introduced some inaccuracies in estimate values, but differences between sites should have been preserved because this method was applied at all sites.

Despite these limitations, our data support findings of other studies that identified positive associations between hospital-wide testing per admission and hospital-onset CDI rates.^20–23^ However, our study approached the study question from the patient perspective, identifying and adjusting for other etiologies of diarrhea. An added strength of this study is estimating diarrheal incidence and testing frequency and rates at the patient-location level. This method allowed for a conservative assessment of the significance of observed differences between patient-location types, using each NHSN-mapped location type as an observation in the analysis. This method also allows for location-specific estimates of testing frequency across many location types, which should be useful for modeling estimates of disease in different surveillance or clinical scenarios.

In summary, we quantified the degree to which frequency of testing differs between patient-location types of acute-care hospitals and which patient factors may drive the likelihood to order such tests. Moreover, differences in testing between surveillance sites persisted even after adjusting for patient factors. This difference was nearly identical to the difference in estimated CDI incidence between the 2 surveillance sites. Comparisons of the estimated incidence of CDI between regions may require some insight into differences in testing practice to best interpret such data.

## Supplementary material

For supplementary material accompanying this paper visit https://doi.org/10.1017/ice.2022.205. click here to view supplementary material

## References

[1] Guh AY , Mu Y , Winston LG , et al. Trends in US burden of *Clostridioides difficile* infection and outcomes. N Engl J Med 2020;382:1320–1330. 32242357 10.1056/NEJMoa1910215 PMC7861882

[2] Alcalá L , Martin A , Marin M , et al. The undiagnosed cases of *Clostridium difficile* infection in a whole nation: where is the problem? Clin Microbiol Infect 2012;18:E204–E213. 22563775 10.1111/j.1469-0691.2012.03883.x

[3] Balsells E , Filipescu T , Kyaw MH , Wiuff C , Campbell H , Nair H. Infection prevention and control of *Clostridium difficile*: a global review of guidelines, strategies, and recommendations. J Glob Health 2016;6:020410. 28028434 10.7189/jogh.06.020410 PMC5140074

[4] Davies KA , Longshaw CM , Davis GL , et al. Underdiagnosis of *Clostridium difficile* across Europe: the European, multicentre, prospective, biannual, point-prevalence study of *Clostridium difficile* infection in hospitalised patients with diarrhoea (EUCLID). Lancet Infect Dis 2014;14:1208–1219. 25455988 10.1016/S1473-3099(14)70991-0

[5] Koo HL , Van JN , Zhao M , et al. Real-time polymerase chain reaction detection of asymptomatic *Clostridium difficile* colonization and rising *C. difficile*–associated disease rates. Infect Control Hosp Epidemiol 2014;35:667–673. 24799643 10.1086/676433

[6] Goodenough D , Sefton S , Overton E , et al. Reductions in positive *Clostridioides difficile* events reportable to National Healthcare Safety Network (NHSN) with adoption of reflex enzyme immunoassay (EIA) testing in 13 Atlanta hospitals. Infect Control Hosp Epidemiol 2022;43:935–938. 34236019 10.1017/ice.2021.145

[7] Bauer MP , Notermans DW , Benthem BH van , et al. *Clostridium difficile* infection in Europe: a hospital-based survey. Lancet 2011;377:63–73. 21084111 10.1016/S0140-6736(10)61266-4

[8] Kamboj M , Brite J , Aslam A , et al. Artificial differences in *Clostridium difficile* infection rates associated with disparity in testing. Emerg Infect Dis 2018;24:584–587. 29460760 10.3201/eid2403.170961 PMC5823336

[9] Alcalá L , Reigadas E , Marín M , et al. Impact of clinical awareness and diagnostic tests on the underdiagnosis of *Clostridium difficile* infection. Eur J Clin Microbiol Infect Dis 2015;34:1515–1525. 25904126 10.1007/s10096-015-2380-3

[10] Curren EJ , Lutgring JD , Kabbani S , et al. Advancing diagnostic stewardship for healthcare-associated infections, antibiotic resistance, and sepsis. Clin Infect Dis 2022;74:723–728. 34346494 10.1093/cid/ciab672

[11] Lessa FC , Mu Y , Bamberg WM , et al. Burden of *Clostridium difficile* infection in the United States. N Engl J Med 2015;372:825–834. 25714160 10.1056/NEJMoa1408913 PMC10966662

[12] Mawer D , Byrne F , Drake S , et al. Cross-sectional study of the prevalence, causes and management of hospital-onset diarrhoea. J Hosp Infect 2019;103:200–209. 31077777 10.1016/j.jhin.2019.05.001

[13] White NC , Mendo-Lopez R , Papamichael K , et al. Laxative use does not preclude diagnosis or reduce disease severity in *Clostridioides difficile* infection. Clin Infect Dis 2019;71:1472–1478. 10.1093/cid/ciz978 PMC7486840 31584632

[14] Polage CR , Solnick JV , Cohen SH. Nosocomial diarrhea: evaluation and treatment of causes other than *Clostridium difficile* . Clin Infect Dis 2012;55:982–989. 22700831 10.1093/cid/cis551 PMC3657522

[15] Cummings P. Missing data and multiple imputation. JAMA Pediatr 2013;167:656–661. 23699969 10.1001/jamapediatrics.2013.1329

[16] Martin D , Austin H. An efficient program for computing conditional maximum likelihood estimates and exact confidence limits for a common odds ratio. Epidemiology 1991;2:359–362. 1742385 10.1097/00001648-199109000-00008

[17] Rock C , Pana Z , Leekha S , et al. National Healthcare Safety Network laboratory-identified *C. difficile* event reporting: a need for diagnostic stewardship. Am J Infect Control 2018;46:456–458. 29305285 10.1016/j.ajic.2017.10.011 PMC6734925

[18] Gould CV , Edwards JR , Cohen J , et al. Effect of nucleic acid amplification testing on population-based incidence rates of *Clostridium difficile* infection. Clin Infect Dis 2013;57:1304–1307. 23899677 10.1093/cid/cit492 PMC9580544

[19] Buckel WR , Avdic E , Carroll KC , Gunaseelan V , Hadhazy E , Cosgrove SE. Gut check: *Clostridium difficile* testing and treatment in the molecular testing era. Infect Control Hosp Epidemiol 2015;36:217–221. 25633006 10.1017/ice.2014.19

[20] Balsells E , Shi T , Leese C , et al. Global burden of *Clostridium difficile* infections: a systematic review and meta-analysis. J Glob Health 2019;9:010407. 30603078 10.7189/jogh.09.010407 PMC6304170

[21] Angulo FJ , Oliv SP , Carrico R , et al. Frequency of stool specimen collection and testing for *Clostridioides difficile* of hospitalized adults and long-term care facility residents with new-onset diarrhea in Louisville, Kentucky. Int J Infect Dis 2022;120:196–200. 35477052 10.1016/j.ijid.2022.04.046

[22] Howard-Anderson JR , Sexton ME , Robichaux C , et al. The impact of an electronic medical record nudge on reducing testing for hospital-onset *Clostridioides difficile* infection. Infect Control Hosp Epidemiol 2020;41:411–417. 32036798 10.1017/ice.2020.12 PMC7909614

[23] Kamboj M , Brite J , Aslam A , et al. Artificial differences in *Clostridium difficile* infection rates associated with disparity in testing. Emerg Infect Dis 2018;24:584–587. 29460760 10.3201/eid2403.170961 PMC5823336

