# Supplementary material for: Determinates of Clostridioides difficile infection (CDI) testing practices among inpatients with diarrhea at selected acute-care hospitals in Rochester, New York, and Atlanta, Georgia, 2020–2021
Source: Infect Control Hosp Epidemiol. 2022 Sep 14;44(7):1085–92. doi: 10.1017/ice.2022.205 (PMC10369210; doi:10.1017/ice.2022.205)

**Supplemental Materia**

Determinates of CDI testing practices among inpatients with diarrhea at selected acute care hospitals in Rochester, New York and Atlanta, GA, 2020-2021.

Supplementary Tables

Table S1: Diarrheal episodes and new diarrhea episodes among hospitalized patients at two study sites, 2021

| Study Site | Activity Time | Facility | Diarrheal episodes | New Diarrheal episodes | Diagnostic Stewardship* |
| --- | --- | --- | --- | --- | --- |
| Site A | Nov, 2020 and  May, 2021 | Hosp. 1 | 420 | 220 | Rejection policy  Laxative alerts  Nurse driven collection  NAAT screen, reflex to EIA if + |
|  |  | Hosp. 2 | 163 | 117 |  |
|  |  | Hosp. 3 | 140 | 98 |  |
|  |  | Total | 723 | 435 |  |
| Site B | Oct-Nov, 2020 and  May-Jun, 2021 | Hosp. 1 | 676 | 318 | Rejection policy  Laxative alerts, requiring approval to test  Nurse education  EIA/GDH screen, reflex to NAAT if + |
|  |  | Hosp. 2 | 189 | 107 |  |
|  |  | Total | 865 | 425 |  |
| Overall |  | Total | 1,588 | 860 |  |

* Site A had same policies in place for all hospital sites. Rejection policy was to not test formed stool or if there has been a negative test w/in past 7 days or positive test w/in past 14 days. Laxative alerts were an electronic health record pop-up advising providers to avoid ordering CDI testing on patients receiving laxatives. Site B allowed providers to over-ride the prompt, while Site A required a phone call to receive permission to order. Nurse education about appropriate testing occurred at both Site A and B, but at site A there was active encouragement for nursing staff to collect diarrheal stools of symptomatic patients on first 3 days of admission. NAAT was initial test at Site A, and a reflex test for positive specimens by EIA. At Site B both facilities had similar testing algorithm of initial EIA for GDH and toxin, and reflex testing GDH positive but toxin-negative specimens. However, note, for this analysis any positive test was considered CDI positive patient to simulate the case definition used in the Emerging Infections Program.

Table S2. Characteristics of hospitalized patients with new diarrheal episodes (N=X), Fall 2020 and Spring 2021Period Prevalence Survey

|  | **Study Site A** | | | **Study Site B** | | **Total   (N=860)** |
| --- | --- | --- | --- | --- | --- | --- |
|  | **Hosp. 1**  **(N=220)** | **Hosp. 2**  **(N=117)** | **Hosp. 3**  **(N=98)** | **Hosp. 1**  **(N=318)** | **Hosp. 2**  **(N=107)** |  |
| **Race** |  |  |  |  |  |  |
| (missing) | 27 | 17 | 11 | 16 | 1 | 72 |
| White | 86 (44.6%) | 18 (18.0%) | 55 (63.2%) | 223 (73.8%) | 80 (75.5%) | 462 (58.6%) |
| Black | 102 (52.8%) | 82 (82.0%) | 30 (34.5%) | 63 (20.9%) | 19 (17.9%) | 296 (37.6%) |
| Other | 5 (2.6%) | 0 (0.0%) | 2 (2.3%) | 16 (5.3%) | 7 (6.6%) | 30 (3.8%) |
| **Age (yrs.)** |  |  |  |  |  |  |
| (missing) | 17 | 6 | 2 | 0 | 0 | 25 |
| <50 | 50 (24.6%) | 34 (30.6%) | 17 (17.7%) | 53 (16.7%) | 22 (20.6%) | 176 (21.1%) |
| 50-64 | 82 (40.4%) | 32 (28.8%) | 31 (32.3%) | 96 (30.2%) | 19 (17.8%) | 260 (31.1%) |
| 65-74 | 47 (23.2%) | 20 (18.0%) | 22 (22.9%) | 95 (29.9%) | 21 (19.6%) | 205 (24.6%) |
| 75+ | 24 (11.8%) | 25 (22.5%) | 26 (27.1%) | 74 (23.3%) | 45 (42.1%) | 194 (23.2%) |
| **Had an ostomy** |  |  |  |  |  |  |
| No | 215 (97.7%) | 114 (97.4%) | 98 (100.0%) | 301 (94.7%) | 102 (95.3%) | 830 (96.5%) |
| Yes | 5 (2.3%) | 3 (2.6%) | 0 (0.0%) | 17 (5.3%) | 5 (4.7%) | 30 (3.5%) |
| **Tube Feeding** |  |  |  |  |  |  |
| No | 145 (65.9%) | 71 (60.7%) | 49 (50.0%) | 207 (65.1%) | 89 (83.2%) | 561 (65.2%) |
| Yes | 75 (34.1%) | 46 (39.3%) | 49 (50.0%) | 111 (34.9%) | 18 (16.8%) | 299 (34.8%) |
| **Received Laxatives** |  |  |  |  |  |  |
| No | 120 (54.5%) | 96 (82.1%) | 74 (75.5%) | 106 (33.3%) | 45 (42.1%) | 441 (51.3%) |
| Yes | 100 (45.5%) | 21 (17.9%) | 24 (24.5%) | 212 (66.7%) | 62 (57.9%) | 419 (48.7%) |
| **Received Chemotherapy** |  |  |  |  |  |  |
| No | 204 (92.7%) | 106 (90.6%) | 93 (94.9%) | 311 (97.8%) | 106 (99.1%) | 820 (95.3%) |
| Yes | 16 (7.3%) | 11 (9.4%) | 5 (5.1%) | 7 (2.2%) | 1 (0.9%) | 40 (4.7%) |
| **Diagnosed with COVID19** |  |  |  |  |  |  |
| No | 210 (95.5%) | 103 (88.0%) | 97 (99.0%) | 314 (98.7%) | 100 (93.5%) | 824 (95.8%) |
| Yes | 10 (4.5%) | 14 (12.0%) | 1 (1.0%) | 4 (1.3%) | 7 (6.5%) | 36 (4.2%) |
| **Length of hospitalization** |  |  |  |  |  |  |
| (missing) | 31 | 13 | 6 | 2 | 0 | 52 |
| Median (Q1, Q3) | 14 (6, 26) | 12 (6, 31) | 9 (6, 16) | 13 (8, 24) | 8 (5, 16) | 12 (6, 23) |
| **hospital Location** |  |  |  |  |  |  |
| Wards | 109 (49.5%) | 66 (56.4%) | 64 (65.3%) | 175 (55.0%) | 87 (81.3%) | 501 (58.3%) |
| ICU | 69 (31.4%) | 46 (39.3%) | 27 (27.6%) | 83 (26.1%) | 20 (18.7%) | 245 (28.5%) |
| ONC | 42 (19.1%) | 5 (4.3%) | 7 (7.1%) | 60 (18.9%) | 0 (0.0%) | 114 (13.3%) |
| **Discharge disposition** |  |  |  |  |  |  |
| (missing) | 41 | 16 | 15 | 8 | 1 | 81 |
| Home | 122 (68.2%) | 60 (59.4%) | 52 (62.7%) | 86 (27.7%) | 43 (40.6%) | 363 (46.6%) |
| Hospital | 12 (6.7%) | 10 (9.9%) | 7 (8.4%) | 45 (14.5%) | 15 (14.2%) | 89 (11.4%) |
| LTC/Rehab/SNF | 18 (10.1%) | 8 (7.9%) | 10 (12.0%) | 145 (46.8%) | 40 (37.7%) | 221 (28.4%) |
| Died/Hospice | 27 (15.1%) | 23 (22.8%) | 14 (16.9%) | 34 (11.0%) | 8 (7.5%) | 106 (13.6%) |
| **Resident within Jurisdiction** |  |  |  |  |  |  |
| (missing) | 7 | 5 | 3 | 0 | 0 | 15 |
| Yes | 119 (55.9%) | 85 (75.9%) | 69 (72.6%) | 170 (53.5%) | 84 (78.5%) | 527 (62.4%) |
| No | 94 (44.1%) | 27 (24.1%) | 26 (27.4%) | 148 (46.5%) | 23 (21.5%) | 318 (37.6%) |

Table S3. Relative risk (95% confidence interval) of specific characteristics of cases with new diarrheal episode (N=860) with CDI testing (top section) and characteristics of cases with new diarrheal episode that had a CDI test (N=302) that was positive (N=50) (bottom section): without and with imputation for missing data

|  | **Without imputation for missing data** | | **With Imputation for missing data** | |
| --- | --- | --- | --- | --- |
|  | **Rel. Risk (95% CI)** | **p value** | **Rel. Risk (95% CI)** | **p value** |
| **CDI Testing (302 or 860 with diarrhea)** |  |  |  |  |
| Received Laxatives |  |  |  |  |
| No | *ref* |  | *ref* |  |
| Yes | 0.59 (0.48, 0.74) | <0.001 | 0.59 (0.48, 0.73) | <0.001 |
| Received Chemotherapy |  |  |  |  |
| No | *ref* |  | *ref* |  |
| Yes | 1.17 (0.89, 1.55) | 0.262 | 1.14 (0.88, 1.48) | 0.307 |
| Resident of EIP surveillance area |  |  |  |  |
| Yes | *ref* |  | *ref* |  |
| No | 0.77 (0.62, 0.94) | 0.011 | 0.79 (0.66, 0.95) | 0.014 |
| Admission Location |  |  |  |  |
| Other (non-Critical Care) | *ref* |  | *ref* |  |
| Critical Care | 0.92 (0.73, 1.15) | 0.452 | 0.94 (0.76, 1.17) | 0.595 |
| Oncology | 1.57 (1.25, 1.98) | <0.001 | 1.57 (1.27, 1.94) | <0.001 |
| Study Site |  |  |  |  |
| Site A | *ref* |  | *ref* |  |
| Site B | 0.51 (0.40, 0.63) | <0.001 | 0.50 (0.41, 0.62) | <0.001 |
| **CDI Positivity (50 or 302 tested)** |  |  |  |  |
| Tube Feeding |  |  |  |  |
| No | *ref* |  |  |  |
| Yes | 0.54 (0.27, 1.08) | 0.083 |  |  |
| Hospital-Onset DE |  |  |  |  |
| No | *ref* |  | *ref* |  |
| Yes | 0.58 (0.34, 1.00) | 0.049 | 0.52 (0.31, 0.88) | 0.016 |
| Study Site |  |  |  |  |
| Site A | *ref* |  | *ref* |  |
| Site B | 0.68 (0.38, 1.25) | 0.219 | 0.88 (0.49, 1.56) | 0.652 |

**Imputation:** Missing data (age [2.9%], race [8.4%], hospital-onset diarrhea [2.7%] and residence within jurisdiction [1.7%]) were assumed to be at least missing at random and were addressed by multiple imputation using method of chained equations, with race and age (groups) as a multinomial outcomes and hospital-onset diarrhea status and jurisdiction as binomial outcomes. Imputed values for missing data were predicted by known patient demographic and clinical characteristics. Thirty multiply imputed datasets were constructed (to enhance stability of regression model estimates) and estimates from imputed datasets were pooled to yield average estimates of the complete data model.

Table S4. Age, Race, and gender specific hospitalized *C. difficile* infection rate (per 1000 admissions) among hospitalized patients who are residents of respective EIP catchment areas, July 1 2020 – June 30, 2021, by Site

|  |  |  | **Site A** | | |  | **Site B** | | |  | Site B / Site A |
| --- | --- | --- | --- | --- | --- | --- | --- | --- | --- | --- | --- |
| **Age group** | **Race group** | **Gender** | **Admits** | **CDI + tests** | **CDI Rate** |  | **Admits** | **CDI + tests** | **CDI Rate** |  | Risk Ratio (95% Confidence Interval) |
| 18-44 | Black | Female | 7,467 | 45 | 6.0 |  | 877 | 3 | 3.4 |  | 0.59 (0.20, 1.80) |
| 18-44 | Black | Male | 2,402 | 26 | 10.8 |  | 749 | 4 | 5.3 |  | 0.56 (0.22, 1.40) |
| 18-44 | White | Female | 1,801 | 10 | 5.6 |  | 902 | 2 | 2.2 |  | 0.50 (0.14, 1.77) |
| 18-44 | White | Male | 1,032 | 9 | 8.7 |  | 928 | 2 | 2.2 |  | 0.38 (0.11, 1.34) |
| 18-44 | Other | Female | 488 | 3 | 6.1 |  | 193 | 1 | 5.2 |  | 0.88 (0.16, 4.83) |
| 18-44 | Other | Male | 186 | 2 | 10.8 |  | 196 | 0 | 0.0 |  | 0.0 (0.0, ND) |
| 45-64 | Black | Female | 5,150 | 63 | 12.2 |  | 1,197 | 12 | 10.0 |  | 0.85 (0.50, 1.43) |
| 45-64 | Black | Male | 4,602 | 62 | 13.5 |  | 1,127 | 14 | 12.4 |  | 0.94 (0.58, 1.50) |
| 45-64 | White | Female | 2,017 | 36 | 17.8 |  | 2,018 | 9 | 4.5 |  | 0.40 (0.22, 0.71) |
| 45-64 | White | Male | 2,773 | 37 | 13.3 |  | 2,563 | 18 | 7.0 |  | 0.68 (0.46, 0.99) |
| 45-64 | Other | Female | 261 | 1 | 3.8 |  | 278 | 1 | 3.6 |  | 0.97 (0.24, 3.89) |
| 45-64 | Other | Male | 359 | 3 | 8.4 |  | 288 | 1 | 3.5 |  | 0.56 (0.10, 3.06) |
| >=65 | Black | Female | 5,529 | 68 | 12.3 |  | 1,001 | 12 | 12.0 |  | 0.98 (0.58, 1.65) |
| >=65 | Black | Male | 4,106 | 62 | 15.1 |  | 823 | 7 | 8.5 |  | 0.60 (0.30, 1.22) |
| >=65 | White | Female | 4,775 | 97 | 20.3 |  | 4,829 | 36 | 7.5 |  | 0.53 (0.40, 0.71) |
| >=65 | White | Male | 5,018 | 62 | 12.4 |  | 4,571 | 21 | 4.6 |  | 0.54 (0.37, 0.78) |
| >=65 | Other | Female | 362 | 4 | 11.0 |  | 301 | 2 | 6.6 |  | 0.73 (0.24, 2.28) |
| >=65 | Other | Male | 441 | 2 | 4.5 |  | 284 | 4 | 14.1 |  | 1.71 (0.97, 3.04) |
| All | All | All | 48,769 | 592 | 12.1 |  | 23,125 | 149 | 6.4 |  | 0.62 (0.54, 0.71)* |

Notes: Admits limited to residents of the catchment area of respective Site, Rate is CDI + diarrheal episodes among hospitalized residents per 100 admits; Risk Ratio for each age, race, gender specific stratum is crude calculated based on Taylor series, and All is a Mantel-Hanzel summary rate ratio adjusting for each stratum using OpenEpi, Version 3.

Relative rate (and 95% confidence intervals) of selected characteristics for case with new diarrheal episode (N=860) being CDI Tested (N=302) (figure A) and for CDI test being positive (N=50) (Figure B) among Hospitalized Patients, Fall 2020 and Spring 2021Period Prevalence Survey


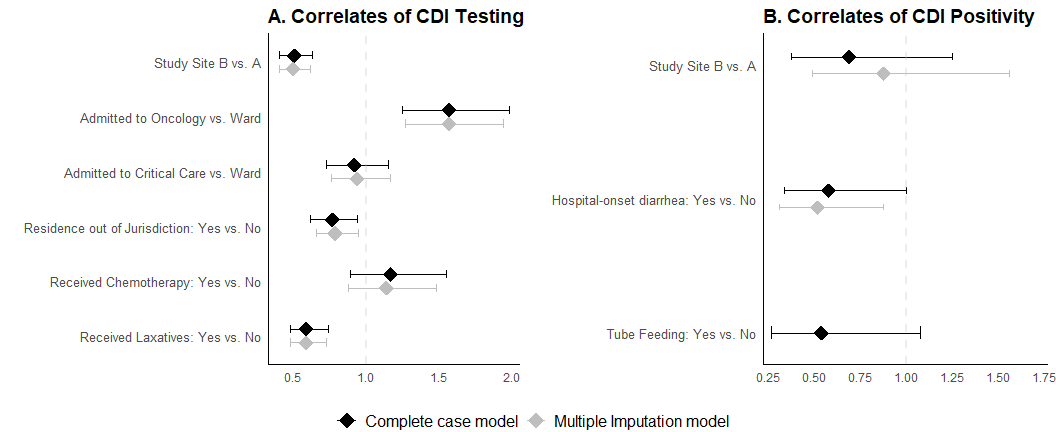

Supplement: Supplementary file 1 [file S0899823X22002057sup001.docx]
